# Habitat Selection by Eld’s Deer following Relocation to a Patchy Landscape

**DOI:** 10.1371/journal.pone.0091158

**Published:** 2014-03-10

**Authors:** Duo Pan, Yan-Ling Song, Zhi-Gao Zeng, Benjamin D. Bravery

**Affiliations:** 1 Key Laboratory of Animal Ecology and Conservation Biology, Institute of Zoology, Chinese Academy of Sciences, Beijing, China; 2 Kexue Communications, Beijing, China; Wildlife Conservation Society, India

## Abstract

An emerging issue in wildlife conservation is the re-establishment of viable populations of endangered species in suitable habitats. Here, we studied habitat selection by a population of Hainan Eld’s deer (*Cervus eldi*) relocated to a patchy landscape of farmland and forest. Hainan Eld’s deer were pushed to the brink of extinction in the 1970s, but their population expanded rapidly from 26 to more than 1000 individuals by 2003 through effective reserve protection. As part of a wider relocation and population management strategy, 131 deer were removed from the reserve and reintroduced into a farmland-forest landscape in 2005. Habitat use under a context of human disturbance was surveyed by monitoring 19 radio-collared animals. The majority of deer locations (77%) were within 0.6–2 km of villages. Annual home ranges of these collared deer averaged 725 ha (SD 436), which was 55% of the size of the reserve from which they had originated. The annual home ranges contained 54% shrub-grassland, 26% forest and 15% farmland. The relocated deer population selected landscape comprising slash-and-burn agriculture and forest, and avoided both intensively farmed areas and areas containing only forest. Within the selected landscape, deer preferred swiddens and shrub-grasslands. Forests above 300 m in elevation were avoided, whereas forests below 300 m in elevation were overrepresented during the dry season and randomly used during the wet season. Our findings show that reintroduced deer can utilize disturbed habitats, and further demonstrate that subsistence agroforest ecosystems have the capacity to sustain endangered ungulates.

## Introduction

Human activity is responsible for a decline of wildlife populations and the deterioration of their habitats across the world [1]. Protected areas established to safeguard wildlife constitute only a small fraction of the Earth’s surface [2], and the spatial limitations placed on protected areas will likely result in management issues in the future. For example, protected areas may be forced to support more animals than is ideal, especially for ungulates that respond well to conservation measures [3]. Further, protected areas may not be effective in restoring species in an ecological sense because limited spatial resources can support only a single or a few populations. Under certain circumstances, maintaining a species solely in a protected area may not reduce its risk of extinction, whereas reintroducing populations outside a protected area can. However, maintaining populations outside reserves and adjacent to human populations poses a unique set of conservation challenges, particularly in relation to the disturbance of wildlife by humans.

Wild animals perceive repeated human disturbance as analogous to predation risk [4], [5]. Human activities increase the heart rates of wildlife, trigger the release of stress hormones and stimulate vigilance behavior [6]–[8]. Human activities can also interrupt normal drinking, foraging and reproductive behavior [5], [9]–[11]. At the individual level, animals respond to repeated human disturbance by allocating extra time to restore their pre-disturbance state; at the population level, the stress of interactions with humans may decrease the reproductive success and survival rates of new-born individuals [12]. Given the impact of human disturbance on the behavior and ecology of wildlife, it is not surprising that many species shift toward areas containing few or no humans [13], [14]. Berger’s ‘risk-disturbance hypothesis’ predicts that a disturbed animal should follow the same economic principles that govern encounters of prey with predators [4], [15], [16]. Avoidance is undoubtedly the most common anti-predator strategy [17]–[19]. However, some ungulates are known to adapt to or even benefit from human activity and seek out human-disturbed areas. For example, endangered key deer (*Odocoileus virginianus clavium*) use urban areas more today than 30 years ago, and the survival rates of deer utilizing urban areas frequently are higher than for deer that utilize urban areas less frequently [20]. Roe deer (*Capreolus capreolus*) use human-disturbed areas as a refuge from lynx (*Lynx lynx*) predation [21]. White-tailed deer (*Odocoileus virginianus*) in North America exploit crops as a food resource, and as such unwanted deer-human interactions arise [22], [23].

Endangered ungulates are candidates for human-dominated ecosystems because of their broad diets and good response to conservation measures. However, they may also damage crops and are often considered a nuisance. Eld’s deer, a medium-sized tropical deer, is an herbivorous generalist patchily distributed across eastern India, Myanmar, Vietnam, Laos, Cambodia and Hainan Island of China. Eld’s deer is endangered in India, Vietnam, Laos, Cambodia and China, and is threatened in Myanmar [24]. On Hainan Island, China, written records regarding Eld’s deer can be traced back to the year 1511. Our lab has previously analyzed variation in mitochondrial DNA D-loop sequences of *C. e. siamensis, C. e. thamin* and *C. e. Hainanus*, and the results suggest that Eld’s deer may have moved via a land bridge from the South-eastern Asian mainland to Hainan Island during the Pleistocene (0.69 Mya ago) [25]. Eld’s deer was once widely distributed on Hainan Island. Because their natural predators were in decline, human hunting drove the collapse of this island population of Eld’s deer and by 1976 only 26 individuals remained, in the region of Datian [26]. Consequently, the Datian Nature Reserve (DNR) was created to specifically protect Eld’s deer in situ. To prevent human disturbances, a fence 2.8 m high was constructed around the perimeter of the 1314-ha protected area. Within the refuge, humans are unable to disturb the deer or deer habitat, except for daily patrols by rangers along regular routes. Eld’s deer here drink water from a natural river and feed on natural vegetation. Wild Indian muntjac (*Muntiacus muntjak nigripes*) and boar (*Sus scrofa chriodontus*) are present as natural competitors, and *Python molurus* as a natural predator [27]. A self-sustaining population of Eld’s deer has thrived in the reserve, recovering to more than 1000 individuals by 2003 [26]. A decision was then made to transfer animals to other parts of the island. According to historical records, Hainan Eld’s deer inhabited flat or gentle slopes below 200 m in elevation covered by forest and grass-shrubs [28], but such habitat has become scarce throughout the island. In 2005, 131 Hainan Eld’s deer were reintroduced to Chihao region, where forests are still numerous, seldom disturbed and mainly distributed at high altitudes. Intensive agricultural land and villages are present at lower altitudes, and slash-and-burn plots and successional stages, such as shrub-grassland, are scattered across the landscape. Eld’s deer went extinct from Chihao in the 1950s.

Because they seldom experience human disturbance or predators, reintroduced ungulates are often naïve and may quickly modify their behavior and habits to adapt to new environments. Behavioral patterns of the reintroduced Eld’s deer at Chihao are consistent with the risk-disturbance hypothesis: deer flee humans upon visual or other contact, and more significantly, shift to a nocturnal foraging pattern to avoid human activity and disturbance [29]. The general effects of reintroduction on deer behavior are well understood. However, ongoing management plans and future strategies for reintroducing this threatened species depend on information about habitat usage and spatial interactions with humans, information that is currently lacking but urgently needed. Specifically, we first need to know what kind(s) of landscape this population chooses. Second, we need to know how individual deer use patches within the selected landscape. Here, we aimed to determine (1) whether deer locations are positively correlated with distance from human villages, and (2) whether deer mainly inhabit forests because they are less exploited and are able to conceal them, or utilize agricultural areas thereby creating spatial overlap and increased interactions with humans.

As reintroductions become a larger part of conservation strategies for a number of key species, and because relatively few empirical studies of human-mediated effects on the distribution of reintroduced populations exist, our results will aid the management and fundamental understanding of animals reintroduced from reserves.

## Study Area and Methods

### Study Area

**Figure 1:**
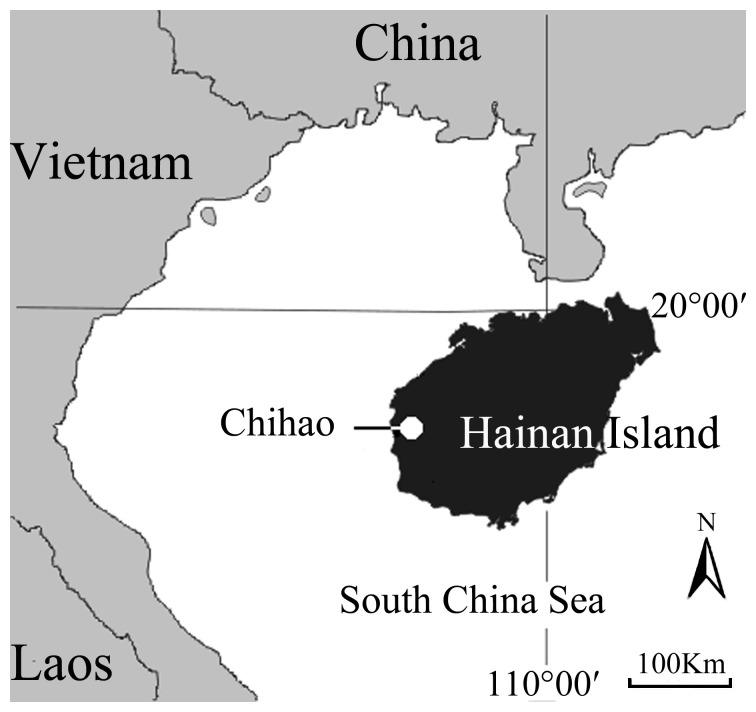
Location of Chihao, Hainan Island, China.

Chihao is located in western Hainan Island, China (108°44′-108°56′E, 18°54′-19°10′N; Fig. 1). The study area is 450 km^2^, with elevation ranging from 10 to 680 m. The climate is hot and dry. The annual average temperature is 24.6°C; July is the hottest month (29.1°C) and January is the coldest (18.4°C). Annual precipitation is 1012 mm, and evaporation is 2522 mm. Most rain occurs in the wet season from July to January.

Chihao habitats are heavily modified. There are 11 villages in the study area with a combined population of 10,000 people. Most residents are Li ethnic minority and indigenous to Hainan. The villagers grow rice, bananas, mangos, rubber trees and eucalyptus. They also raise a small number of goats, cattle and pigs. Hunting is strictly forbidden in the area. Five forest rangers patrol weekly and have occasionally found steel traps for rabbits laid by some older villagers that could accidentally trap deer. Farming, firewood collection and livestock herding occur year round. Beyond a few free-ranging dogs and wild boar, there are no other large or medium-sized mammals in the wild.

The original vegetation is monsoon forest dominated by *Terminalia hainanensis, Albizia odoratissima, Albizia procera, Lannea grandis, Aporosa chinensis, Euphoria longan* and *Diospyros philippensis* [30]. About 35% of the area continues to sustain forest, where is hilly, elevated, and located several kilometers from villages. Agriculture covers 50% of the study area. Rice, bananas and mangos are grown on flat areas and gentle slopes. In hilly areas, small plots of Chinese bean, maize, sweet potato and cassava are created and managed by slashing and burning vegetation. These plots are referred to as ‘swiddens’. After 2 years, fertility declines and the swiddens are abandoned and natural vegetation returns, dominated by *Imperata cylindrica* var. *major*, *Hyptis suavedens*, *Lygodium microstadyum*, *Desodium dunnii*, and *Acacia pennata*. This kind of successional vegetation, described here as ‘shrub-grasslands’, contains the greatest species richness in the study area and is occasionally used for livestock grazing. Across the landscape, there are nine patch types relevant to people and deer (Table 1).

**Table 1:** Description of landscapes.

Land cover	Description
Non-vegetation	
Village	Villages lie in flat and low areas, and include houses and 30-m buffer zones around settlements. Approximately 1000 people live in a village.Most families have dogs, ungulate livestock and fowl.
Water surface	Includes reservoirs and small ponds. Reservoirs are usually about 0.5–1 km away from villages and are used for irrigation, fishing and washing; ponds arescattered throughout the study area and are seldom used by people.
Vegetation	
Paddy field	Often within 2 km of village. Broad in size and intensively farmed to grow rice. Chemical fertilizers and pesticides are applied and irrigation is required.
Dry field	Near villages, broad in size, and used for cultivating bananas and mangos. They are intensively cultured and people often work there.
Swidden	Usually far from villages, small in size and scattered on slopes less than 250 m in elevation. Sugarcane, corn and cassava are grown, managed by rotation.People only appear here during sowing and harvesting seasons.
Plantation	Close to roads and not far from villages. People work here occasionally growing rubber trees and eucalyptus.
Shrub-grassland	Succession from abandoned swidden. Vegetation regrows naturally with rich biodiversity. People use it irregularly to graze a small number of sheep andcattle.
Grassland	Covered by grass less than 1 m tall and dominated by *Eupatorium catarium* and *E. odoratum*, which are unpalatable to ungulates. Local people and theirlivestock casually pass by.
Forest	Farther from villages, occurring on upper parts of hills. Local people collect firewood casually or just pass by.

### Ethical Statement

This work follows an animal-capture protocol (Hudonghan #2004-125) approved by the National Forestry Agency of China. The study was carried out under the authority of a scientific permit issued by the Animal Welfare Committee of the Chinese Academy of Sciences.

### Deer Relocation and Radio Tracking

Male Eld’s deer are larger than females (male, 100 kg; female, 60 kg). Mating occurs during the dry season from February to June. Females give birth in the wet season between late September and early December [31]. The female:male ratio of deer in DNR–all descendants of the original 26 survivors–is 1∶0.7 [32]. They range freely within the enclosure and seldom encounter humans apart from reserve wardens. Deer in DNR were captured at random by encircling with a nylon net. A total of 131 Eld’s deer (79 females and 52 males) were transferred to Chihao from March to July 2005 in six batches; 19 adults (2–8 years old; 10 females and 9 males) were fitted with radio-collar transmitters. Five deer were fitted with MOD-335 (150–152 MHz, 140 g; Telonics, AZ, USA) and released in March 2005, and 14 were fitted with SMRC-1 (151–152 MHz, 160 g; Lotek Wireless Inc., Newmarket, Canada) and released in July 2005.

Collared deer were intensively tracked on foot and by motorcycle in the daytime from July 2005 to November 2006 using a portable receiver (Telonics TR-4) equipped with a three-element Yagi antenna (Telonics). Animal locations were determined by triangulation, and approximately one location was obtained for each collared deer every 3 days.

### Home Range Calculation

ArcView 3.3 (Environmental Systems Research Institute, Redlands, CA, USA) and Home Range Extensions were used to estimate home ranges. The 95% fixed-kernel method with least-squares cross-validation was used to calculate individual annual home ranges [33]–[35]. The study period covered two wet seasons and one dry season: wet^1^ (August–December 2005), dry (February 2006–June 2006) and wet^2^ (August–December 2006). The 100% minimum convex polygon (MCP) method was used to calculate individual seasonal home ranges because the number of observations was less than 50 and the emphasis was on relative size [36].

### Habitat Evaluation and Mapping

Habitat qualities of the seven vegetation patch types (forest, shrub-grassland, swidden, grassland, paddy field, dry field and plantation) were roughly evaluated on the basis of anthropogenic disturbance intensity, edible food abundance and capacity to conceal deer. We interviewed 100 representative local villagers in 2006 to determine the number of days in the previous year they spent in each patch type. We then used the number of activity days per unit area to estimate human disturbance intensity. We collected and weighed the edible parts of grasses, shrubs and trees from 30 (1×1 m) random samples in each patch type to estimate edible food abundance. Hiding cover (concealment) was measured using a 10×130 cm plastic bar, following the method of Griffith and Youtie [37].

We obtained a landscape map based on land cover from the Hainan Forestry Bureau and corrected it by walking along patch boundaries of core areas of deer habitat with a GPS and then mapping it using ArcGIS 9.2 (Environmental Systems Research Institute). We obtained a human disturbance map based on the landscape map by substituting disturbance intensity for each patch type. We also obtained a digital elevation map (DEM; 30-m resolution) from the State Bureau of Surveying and Mapping of China. By overlaying telemetry locations and home ranges on these maps, we were able to analyze habitat use and selection for habitat patch type, elevation and human disturbance.

### Habitat Selection Analysis

**Figure 2:**
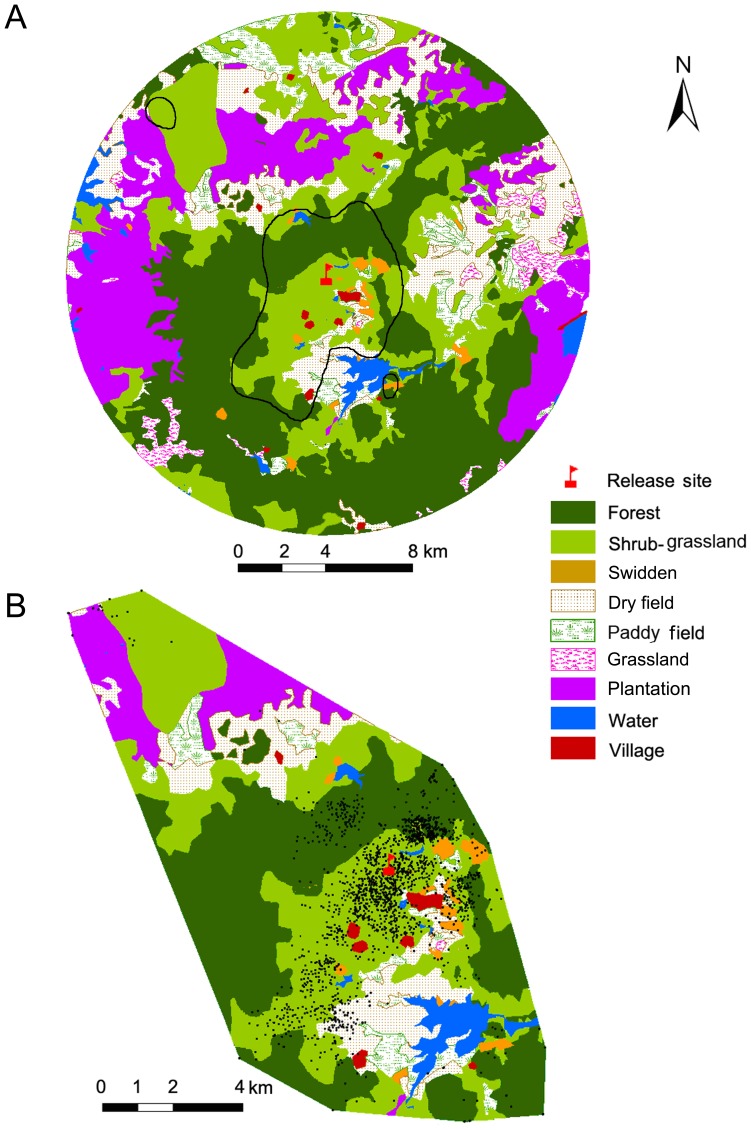
Habitat selection of Eld’s deer on a landscape scale (a) and stand scale (b). (a) The available landscape is indicated by a circle centered on the release site with a radius corresponding to the distance to the farthest location recorded for a collared individual. The used landscape is denoted by the convex polygon (black line) inside the circle, which is the 97% kernel home range calculated from all locations for collared Eld’s deer (n  = 17). (b) All collared deer telemetry locations (black points) and their 100% MCP home range (available habitat) are shown.

Considering that our focus was on group habitat selection, and habitat availability was the same for all reintroduced individuals, we pooled collared individuals for analysis. We asked two questions: (1) what type of landscape do reintroduced deer select within the study area, and (2) what patches do animals prefer within their selected landscape? To answer the first question, we represented the available habitat as a circle with its center at the release site and a radius corresponding to the distance of the farthest recorded location of a collared individual. We defined the 97% kernel group home range as ‘used landscape’ (Fig. 2a). To answer the second question, we defined 100% MCP group home ranges as available habitat, and used the numbers of telemetry locations in each patch category to evaluate patch use (Fig. 2b).

We performed a log-likelihood 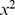 test to determine whether deer selectively used habitat initially [38]:
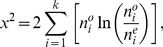
(1)where *k* is the number of habitat categories, 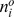 is the quantity of habitat patch category *i* used by deer, and 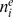 is the expected quantity of habitat patch category *i* used. The null hypothesis is that deer used each patch category in proportion to its relative abundance (randomly used). If the null hypothesis is rejected, at least one habitat experienced significant selection. We then determined which habitats were selected by applying the Manly-Chesson selectivity index (2) and Bonferroni-adjusted 95% confidence intervals (3) [38] to obtain the selectivity index 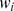 as
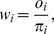
(2)where *O_i_* is the proportion of habitat *i* used, and *π_i_* is the proportion of habitat *i* available. For landscape-scale selection, habitat is preferred if the selectivity index is >1 and avoided if <1,
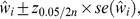
(3)where *n* is the total number of comparisons being made. For stand-scale selection, habitat category i is preferred if the interval is >1 and avoided when <1. If the confidence interval includes 1, the habitat type is randomly used. The standard error (4) is
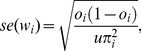
(4)where *u* is the total number of telemetry locations.

### Statistical Analysis

Values are expressed as means ± standard deviations. All statistical analyses were performed using SPSS 18.0. Pearson correlation analyses were used to describe relationships between the number of telemetry locations and the distance from villages, as well as the relationship between annual home range size and the number of habitat categories it contained. Mann-Whitney U-tests were used to compare differences in home range size between seasons. Habitat use in wet^1^ season was influenced by the release of the animals, so only data from the dry and wet^2^ seasons were used in χ^2^ tests.

## Results

### Overview of Location and Habitat

Two collared deer died very early in the study, one from intestinal obstruction and one during a typhoon. In addition: one deer (33F2) lost its collar; the batteries failed in the collars of three deer (36F5, 76F6 and 96M4); and the collar of one deer (71F2) remained undetected from January to March 2006. Ultimately, 12 individuals (6 females and 6 males) were monitored over an entire year, and their locations were used to analyze home range and habitat use. Five collared individuals (3 females and 2 males) were monitored for less than a year, and their locations were only used to analyze habitat selection (Table 2).

**Table 2:** Deer locations used for calculating home range and analyzing habitat use.

Collareddeer	Release time(mm-yy)	Stop monitoring time(mm-yy)	Totallocations	Locations for annualhome range*	Locations for seasonalhome ranges (Wet^1^/Dry/Wet^2^)	Locations for annualhabitat use**	Locations for seasonal habitat use(Dry/Wet)
01F5	07-05	11-06	139	93	40/32/30	89	30/30
16F4	03-05	07-06	107	92	37/32/–	92	31/34
21F5	07-05	12-06	149	89	40/31/33	86	31/33
23F5	07-05	12-06	91	58	22/20/11	57	20/11
36F5	03-05	12-05	52	–	40/–/–	–	–/36
41F6	07-05	12-06	155	93	40/34/32	87	34/32
51F3	07-05	12-06	147	95	39/34/33	94	34/33
71F2	07-05	12-06	92	–	24/–/31	–	–/31
76F6	03-05	04-06	77	–	35/–/–	–	–/32
11M8	07-05	11-06	129	100	23/35/33	95	34/31
13M2	07-05	11-06	133	88	40/30/27	85	19/19
31M2	07-05	12-06	158	98	40/33/33	96	30/32
33F2	07-05	11-05	54	–	–	–	–
43M2	07-05	12-06	157	94	40/31/33	90	31/33
61M4	07-05	12-06	146	97	40/35/33	93	33/33
91M7	07-05	12-06	124	84	30/30/29	83	30/29
96M4	03-05	03-06	76	–	39/–/–	–	–/36

*To minimize the effect of release on annual home range, we used locations between November 2005 and November 2006 for animals released in July 2005 (July 2005–July 2006 for 16F4).

**Locations within 100 m of the boundary of patches were omitted since there is uncertainty associated with locations obtained by triangulation.

In the Chihao region (CHR), human activity intensity is negatively related to elevation. Forests are mainly distributed at higher altitudes (mean = 272 m) and are the least disturbed landscape. Because forests also provide good concealment, the forest is the safest of all patch types for deer. Among the three medium level disturbed patch types, shrub-grassland offered the best quality by virtue of its good food abundance and medium concealment. Swiddens were often adjacent to shrub-grasslands or embedded in them. Intensive agricultural areas were all heavily distributed and occupied lower altitudes (mean  = 94 m) (Table 3).

**Table 3:** Habitat quality across the study area.

Vegetation type	Human disturbance intensity(labor days/ha/year)	Edible food abundance(g/m^2^)	Concealment condition(% of cover)	90% area elevationrange (m)
Forest	Weak (<10)	Medium (100–200)	Good (>80)	150–520
Shrub-grassland	Medium (11–40)	Good (>200)	Medium (60–80)	50–220
Swidden	Medium (11–40)	Good (>200)	Poor (<60)	125–210
Grassland	Medium^a^ (11–40)	Medium (100–200)	Poor (<60)	30–150
Paddy field	Heavy (>40)	Good (>200)	Poor (<60)	50–160
Plantation	Heavy^b^ (>40)	Medium (100–200)	Good (>80)	30–150
Dry field	Heavy (>40)	Poor (<100)	Medium (60–80)	40–160

a People or livestock pass through occasionally; disturbance duration is short.

b Close to traffic; disturbance is heavy.

### Spatial Distribution of Eld’s Deer

**Figure 3:**
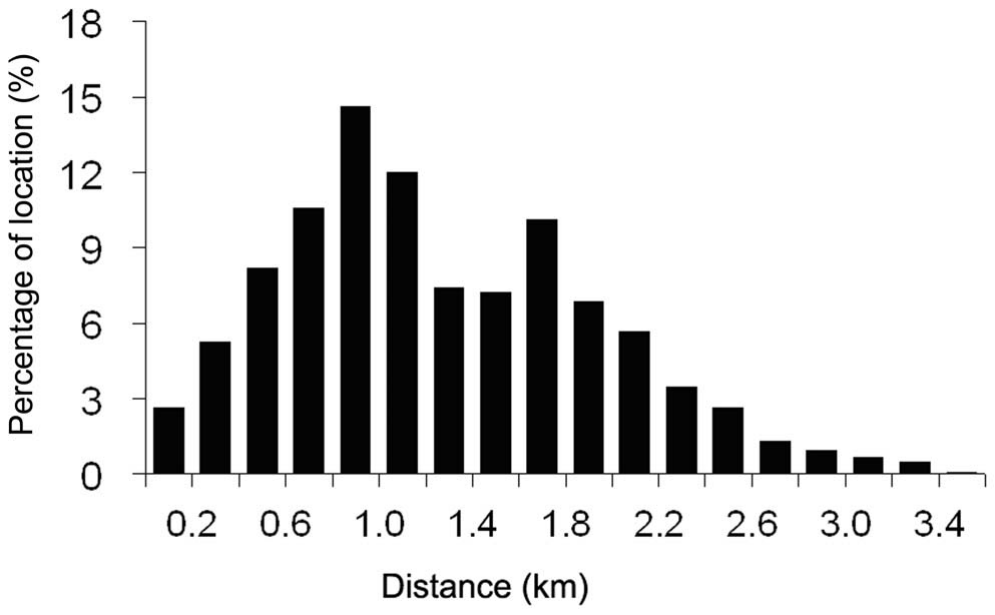
Distances between relocated Eld’s deer and the nearest village.

Although our recordings showed that Eld’s deer could travel up to 3.3 km (straight-line distance) within a single day, the average distance from a known Eld’s deer location to the nearest village was only 1.3 km. The majority of deer locations (77%) were between 0.6–2 km from villages. Only 5% of locations were farther than 2.4 km from villages (Fig. 3). Within 1 km of villages, the number of deer locations (32% of total) was positively correlated with distance from the village (r = 0.996, *p*<0.001); however, this correlation was negative at distances greater than 1 km from a village (r = −0.932, *p*<0.001).

### Annual and Seasonal Home Ranges

The average annual home range for a reintroduced deer was 725±436 ha (n = 12), which is 55% of the size of the reserve from which they originated. Annual home range was highly variable between animals (range = 137–1384 ha). The seasonal home ranges were 483±225 ha (range = 241–883 ha; n = 10) in wet^1^ season, 698±624 ha (range = 72–1920 ha; n = 11) in the dry season, and 272±158 ha (range = 57–531 ha; n = 10) in wet^2^ season. Home ranges in wet^1^ season were significantly larger than those in wet^2^ (Z = 2.269, *p* = 0.023). Home ranges in the dry season trended towards being larger than those in the two wet seasons, but differences were not significant (Z = 0.211, *p* = 0.833; Z = 1.620, *p* = 0.105).

### Habitat Types within Home Ranges

**Figure 4:**
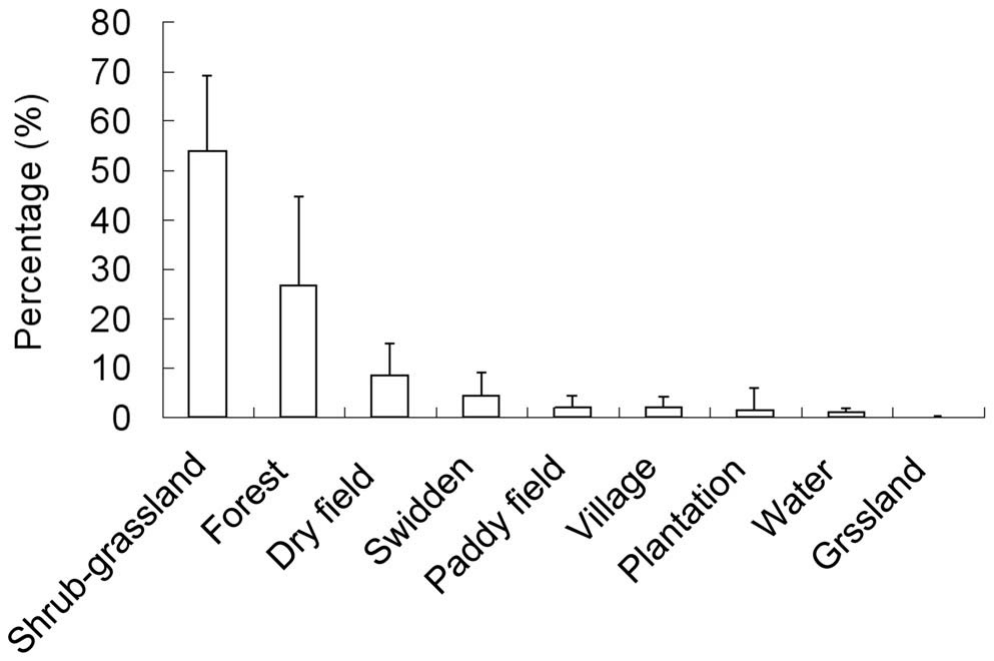
Average percentage of each patch category within the home range of an Eld’s deer.

The annual home range of each collared deer contained four to nine patch types. There was a positive relationship between home range size and the number of patch types (r = 0.534, *p* = 0.09; n = 12). Shrub-grassland, forest, swidden and water were features of all 12 collared deer home ranges. Although the dry field category composed a larger proportion of the annual home range than swidden on average (Fig. 4), it was found in the home ranges of nine individuals. Two other heavily disturbed patch categories, paddy field and village, were included in the home ranges of eight and four animals, respectively. Grassland was contained within the home range of one animal and plantation was found in the home range of two animals. On average, shrub-grasslands constituted the majority of home ranges (54.1%), followed by forest (26.4%) and farmland (dry field, swidden and paddy field; 15% in total) (Fig. 4).

### Habitat Selection

#### Landscape scale

At the landscape scale, deer preferred areas above 150 m in elevation and avoided areas below 150 m in elevation. Medium and weakly disturbed patches were overrepresented and heavily disturbed areas were underrepresented. Deer showed a clear preference for shrub-grassland and swidden, and a very slight preference for forest. Surprisingly, villages were also overrepresented at this scale. Deer exhibited an order of avoidance of grassland>plantation>paddy field> water> dry field (Table 4). These patterns suggest that Eld’s deer inhabit areas comprising traditional agriculture and forest. They avoided both landscapes consisting of only forest and those dominated by intensive farmland (Table 4; Fig. 2a).

**Table 4:** Habitat selection by reintroduced Eld’s deer at a landscape scale.

Habitat variable	Type	*π_i_*	*o_i_*	*w_i_*
Elevation (m)	<75	0.252	0.022	0.09–
	75–150	0.346	0.161	0.47–
	150–225	0.209	0.486	2.33+
	225–300	0.098	0.170	1.74+
	300–375	0.050	0.082	1.65+
	375–450	0.025	0.041	1.66+
	450–680	0.021	0.037	1.75+
	Total	1.000	1.000	9.69
Human disturbance	Weak	0.353	0.408	1.16+
	Medium	0.278	0.409	1.48+
	Heavy	0.368	0.183	0.50–
	Total	1.000	1.000	3.14
Land cover	Forest	0.346	0.352	1.02
	Shrub-grassland	0.241	0.454	1.88+
	Swidden	0.007	0.034	4.85+
	Dry field	0.128	0.093	0.73–
	Paddy field	0.055	0.025	0.45–
	Grassland	0.024	0.001	0.05–
	Plantation	0.178	0.010	0.06–
	Water	0.018	0.011	0.63–
	Village	0.004	0.019	4.80+
	Total	1.000	1.000	14.41

Habitat was preferred for *w_i_*>1 (+) and avoided for *w_i_*<1 (–).

#### Stand scale

Within the selected landscape (100% MCP group home range), reintroduced deer selectively used habitats with different altitudes (χ^2^ = 1903.7, df = 6, p<0.001), patch categories (χ^2^ = 1136.7, df = 8, p<0.001) and disturbance (χ^2^ = 7127.3, df = 2, p<0.001).

At this scale, only areas at elevations of 150–225 m were overrepresented. Elevations of 225–300 m were used in proportion year round, and areas below 150 m and above 300 m in elevation were definitely avoided (Table 5). χ^2^ test showed no significant difference in elevation use between seasons (*p*>0.1).

**Table 5:** Habitat selection by reintroduced Eld’s deer at the stand scale.

Habitat variable	Type		Entire tracking	period	Dry	season	Wet	season
		*π_i_*	*o_i_*	*w_i_*	*o_i_*	*w_i_*	*o_i_*	*w_i_*
Elevation (m)	<75	0.206	0.010	0.05–	0.008	0.04–	0.000	0.00–
	75–150	0.153	0.081	0.53–	0.115	0.75–	0.074	0.48–
	150–225	0.343	0.748	2.18+	0.707	2.06+	0.754	2.20+
	225–300	0.137	0.151	1.11	0.148	1.08	0.153	1.12
	300–375	0.077	0.010	0.13–	0.022	0.29–	0.017	0.22–
	375–450	0.042	0.001	0.02–	0.000	0.00–	0.002	0.06–
	450–680	0.043	0.000	0.00–	0.000	0.00–	0.000	0.00–
	Total	1.000	1.000	4.02	1.000	4.22	1.000	4.08
Human disturbance	Weak	0.368	0.225	0.61–	0.288	0.78–	0.196	0.53–
	Medium	0.380	0.705	1.86+	0.615	1.62+	0.699	1.84+
	Heavy	0.252	0.066	0.26–	0.096	0.38–	0.105	0.42–
	Total	1.000	1.000	2.73	1.000	2.78	1.000	2.79
Land cover	Forest >300 m	0.169	0.012	0.07–	0.022	0.13–	0.019	0.11–
	Forest ≤300 m	0.199	0.212	1.07	0.266	1.34+	0.177	0.89
	Shrub-grassland	0.360	0.678	1.88+	0.585	1.62+	0.663	1.84+
	Swidden	0.019	0.030	1.60+	0.030	1.60+	0.036	1.92+
	Dry field	0.124	0.051	0.41–	0.058	0.47–	0.095	0.77
	Paddy field	0.046	0.013	0.28–	0.036	0.79	0.010	0.22–
	Grassland	0.001	0.000	0.00–	0.000	0.00–	0.000	0.00–
	Plantation	0.082	0.003	0.04–	0.003	0.04–	0.000	0.00–
	Water*	–	–	–	–	–	–	–
	Village*	–	–	–	–	–	–	–
	Total	1.000	1.000	5.35	1.000	5.99	1.000	5.75

*No locations were within these patch types, so they were considered unavailable.

Habitat was preferred for confidence interval *w_i_*>1 (+) and avoided for *w_i_*<1 (–). Habitats without markings were randomly used.

Deer preferred medium-disturbed habitats throughout the year; both weakly and heavily disturbed habitats were avoided (Table 5). Areas suffering different disturbance levels were used differently across the seasons (χ^2^ = 9.234, df = 2, *p* = 0.01). For example, deer used medium-disturbed habitats more and low-disturbed habitats less during the wet season than the dry season.

Deer showed a preference for shrub-grasslands and swiddens throughout the year. Two-thirds of their locations were distributed within shrub-grasslands (Table 5; Fig. 2b). Forest above 300 m in elevation was avoided all year round. Forest below 300 m in elevation was overrepresented during the dry season and was randomly used in the wet season. Approximately 20–29% of locations were in forest. Regarding intensively cultured land, deer randomly used paddy fields in the dry season and dry fields in the wet season. Plantations, grassland and villages were avoided all year (Table 5). Habitat use was different between the dry and wet seasons (χ^2^ = 19.5, df = 4, *p* = 0.001). For example, deer used shrub-grasslands more frequently and forests less frequently during the wet season than during the dry season.

## Discussion

Our results show that reintroduced deer were active around villages that maintained traditional agricultural practices. The distance of reintroduced deer from villages was small, not only compared with their daily movements, but also compared with the distances that other populations or deer species maintain from infrastructure. For example, a comprehensive analysis of Eld’s deer populations in Cambodia, Myanmar, Lao, Thailand and Vietnam showed that they were more likely to be found at a greater distance from human settlements and more than 10 km from the nearest village [39]. A similar pattern of mule deer (*Odocoileus hemionus*) distribution around drilling wells was also found: although a single well pad typically disturbs 1.2–1.6 ha of habitat, areas with the highest probability of use by female mule deer were 2.7, 3.1 and 3.7 km away from well pads during years 1, 2 and 3 after development, respectively [40].

Presumably, Eld’s deer could inhabit areas close to humans at Chihao because they had not experienced human disturbance while living in the reserve, except during antler harvest days in October. Stankowich [41] found that animals subjected to greater hunting activity are more wary. If animals seldom experience human hunting, they do not treat humans as threats and develop less fear [42], [43]. Further, better quality food (crops and fresh plant growth in fallows) is associated with proximity to humans; thus, animals were attracted to populated areas. Spatial heterogeneity was high in Chihao, even in areas near villages, so Eld’s deer can conceal themselves in these heterogeneous microhabitats.

Our evaluation of habitat selection was guided by the framework developed by Johnson [44] and the scales in our study basically corresponded to the second- and third-order selection [44]. In our analysis, we did not strictly follow Johnson’s method because Eld’s deer are not territorial; their individual home ranges are their scope of movement, not the space they defend. Accordingly, we did not use individual home ranges when evaluating habitat selection. The process used to evaluate habitat selection is often a source of controversy. To strengthen our conclusions, we used several different methods–MCP 96 to 99 and kernel 95 to 99–to estimate the used landscape at a large scale. The results from these methods showed similar trends: shrub-grasslands and swiddens were preferred; forests were very slightly preferred or used randomly; and grasslands, plantations and intensively cultured fields were avoided. Villages were overrepresented because villages in Chihao are small (<50 ha each) and deer locations were close to villages, making it easy to include this patch type in used landscapes using the kernel or MCP method. Only selection for water was affected by the method used: it was preferred if the used landscape was estimated by MCP 98, MCP 99 and kernel 99; randomly used according to kernel 98; and avoided according MCP 96, MCP 97 and kernel 95–97. This variation reflected the fact that used landscape boundaries crossed the largest reservoir in the study area.

The selection index for shrub-grassland was higher than that for forest at both scales, and home ranges contained more shrub-grassland than forest. Plants in shrub-grasslands are new growth, and deer are well known to prefer fresh and tender grasses and shrubs [45]. Given that two-thirds of deer feeding activity occurs during the night [29], shrub-grasslands were likely to be used even more frequently than revealed by our data, which were collected during the day. By contrast, the three subspecies of Eld’s deer in Cambodia, Myanmar, Lao, Thailand and Vietnam all occupy open forests. *C. e. thamin* are most often found in dry, deciduous dipterocarp forests with an open understory in central Myanmar [39], [46]. These differences in habitat selection are probably attributable to the fact that Eld’s deer on Hainan Island were not very sensitive to humans, and were able to withstand certain human disturbances to obtain high quality food in shrub-grasslands and swiddens. Also, traditional agriculture lands were closer to water than forests. Similarly, Eld’s deer at another reintroduced site (Houmiling Nature Reserve) show a preference for habitat near water [47]. In addition, forests in Chihao often occupy hills above 250 m in elevation and McShea et al. reported detecting no Eld’s deer populations at elevations above 400 m [39], [48].

Although forests offer better concealment, ample forage and are the least disturbed patch type, relocated deer selected medium-disturbed areas where high quality food and water were readily attainable. This is contrary to predictions from the ‘risk-disturbance hypothesis’, which posits that animals prefer habitats away from human activity or infrastructure, even if these areas offer poorer grazing conditions [49], [50]. Roe deer in modified landscapes retain strong links to woodland structures if woodland fragments are numerous and widely dispersed, but they adopt an open field habit where the remaining woodland is clumped, with little edge [51]. Both roe deer and Eld’s deer show behavioral plasticity and ecological flexibility, which enables them to make use of secondary matrix habitats. By fleeing approaching people and remaining concealed during times of peak human activity [29], but withstanding certain human activities, they are able to exploit resources and optimize their fitness. This indicates that avoidance is not the only principle that guides the behavior of wild ungulates. If human activities are non-fatal, there will be tradeoffs and potential benefits from closer association.

In the dry season, vegetation concealment, food and water deteriorated. Deer used forest more frequently, as it is the safest patch type, and visited paddy fields more for food and water. Additionally, Eld’s deer enter estrus and seek mates in the dry season [52]. Therefore, the home ranges of Eld’s deer were larger in the dry season than in the wet season, in agreement with a study on *C. e. thamin*, another subspecies of Eld’s deer in the Chatthin Wildlife Sanctuary of Myanmar [53]. Comparisons between the two wet seasons indicated that efforts to locate suitable habitat during the early stages of reintroduction might result in increased deer movement.

The annual home range size of *C. e. hainanus* (7.3 km^2^) is similar to that of *C. e. thamin* (8 km^2^) in Myanmar. Human activity in the Chatthin Wildlife Sanctuary impacts deer to a lesser degree [48], [53]; here, human disturbance and habitat differences do not appear to have reduced or enlarged home range sizes overall. However, in Myanmar, the annual home range of females is smaller than males, a pattern consistent across a number of taxa [54]–[56]. At Chihao, the home range of females was larger than that of males. Possible differences in the ways male and female *C. e. hainanus* respond to anthropogenic environments will be the subject of our next study.

## Management Implications

Our results provide managers with practical information on the reintroduction of Eld’s deer to a patchy landscape. The reintroduced Eld’s deer chose to inhabit landscapes comprising traditional agriculture and forest. These findings provide a reminder that preservation of traditional cultivation can be very important for deer survival but that crop raiding by Eld’s deer will be inevitable. To reduce human-deer conflict and assure successful re-establishment of viable populations, we recommend that reintroduced populations be co-managed by the DNR and local communities. Community co-management is helpful in controlling anthropogenic disturbances to Eld’s deer, especially during their rutting and breeding periods. Although forests were not preferred by deer as shrub-grasslands were, they appeared in each focal animal’s home range. Because forests are the least disturbed habitat, they might act as corridor for dispersal of deer into and across agricultural areas [57], and provide adequate safety during pregnancy, calving and antler growth [58]–[60]. Our results support the idea that large sections of forest are predictive of Eld’s deer presence [39], and suggest that maintaining forested areas is essential to the future recovery of this species. In addition, collared deer had a large annual home range (up to 1384 ha), indicating that Eld’s deer in the fenced DNR have restricted movement and spatial behavior. Although the fenced DNR allowed for the increase in population size, it should be enlarged by incorporating circumjacent regions with low disturbance and good habitat conditions.
